# Influence of COVID-19 on postoperative prognosis and pain management

**DOI:** 10.1371/journal.pone.0344211

**Published:** 2026-03-09

**Authors:** Yue-Zi Hu, Zai-Long Qin, Wen Tang, Zhao-Lan Hu, Ru-Yi Luo

**Affiliations:** 1 Clinical Laboratory, The Second Affiliated Hospital of Hunan University of Chinese Medicine, Changsha, Hunan, China; 2 Guangxi Key Laboratory of Reproductive Health and Birth Defect Prevention, Maternal and Child Health Hospital of Guangxi Zhuang Autonomous Region, Nanning, Guangxi, China; 3 Guangxi Clinical Research Center for Birth Defects, Maternal and Child Health Hospital of Guangxi Zhuang Autonomous Region, Nanning, Guangxi, China; 4 Department of Anesthesiology, The Second Xiangya Hospital, Central South University, Changsha, Hunan, China; Ataturk University Faculty of Medicine, TÜRKIYE

## Abstract

**Background:**

The COVID-19 pandemic has significantly affected healthcare, particularly surgical care. Although short-term effects on surgical outcomes have been examined, understanding of long-term postoperative prognosis and pain management in COVID-19 patients remains limited. This knowledge gap is critical as the pandemic evolves and the need for optimized postoperative care becomes increasingly important.

**Objective:**

The primary objective of this study was to evaluate the impact of COVID-19 infection on postoperative outcomes and pain management in surgical patients. We aimed to assess surgical mortality, complication rates, and postoperative pain levels in COVID-19-positive patients relative to a closely matched control group.

**Methods:**

We conducted a retrospective cohort study of COVID-19 patients admitted to the ICU following surgery. Data were collected on baseline characteristics, postoperative complications, mortality and pain scores. Univariate and multivariate linear regression models were used to evaluate the impact of COVID-19 infection on postoperative pain. Stratified and interaction analyses were additionally performed to examine the robustness of these associations across subgroups.

**Results:**

Mortality rates and the incidence of sepsis were significantly higher in the COVID-19 cohort. Patients with COVID-19 also experienced longer duration of mechanical ventilation in the ICU and prolonged ICU stays. In the fully adjusted multivariate linear regression model, COVID-19 infection was positively associated with higher postoperative visual analog scale pain scores (β = 1.51; 95% CI: 1.03–1.98; p < 0.001), corresponding to an average increase of 1.51 units in postoperative pain. Stratified analysis largely corroborated these findings across subgroups.

**Conclusions:**

Surgical intervention in patients with COVID-19 was associated with higher mortality and sepsis rates, longer ICU stays, and increased postoperative pain scores. These findings highlight the need for continued research to optimize surgical care and improve patient outcomes in the evolving post-pandemic era.

## Introduction

The coronavirus disease 2019 (COVID-19) pandemic has placed significant strain on healthcare systems worldwide and has led to considerable economic losses [1]. Global adult mortality rates surged during the pandemic in 2020 and 2021, reversing the previously observed decline [2]. The primary clinical manifestations of COVID-19 include fever, dry cough, dyspnea, myalgia, fatigue, lymphopenia, and radiographic evidence of pneumonia [3]. For anesthesiologists, fever or pneumonia is considered a relative contraindication for elective surgery. A 2024 report in The Lancet indicated that COVID-19 was the leading cause of disability-adjusted life-years (DALYs) globally, followed by ischemic heart disease, neonatal disorders, and stroke [4]. Consequently, although the peak of the COVID-19 epidemic has subsided, the virus continues to impose a significant medical burden, and surgical patients infected with COVID-19 remain a clinical concern. However, with the establishment of standardized COVID-19 management protocols, routine preoperative testing is no longer universally performed. Thus, the effects of COVID-19 on surgical patients remain insufficiently understood.

Research has demonstrated that the risks associated with elective and emergency surgery in individuals infected with COVID-19 are significantly elevated [5]. One study reported higher surgical mortality and complication rates in patients with COVID-19 compared to those without the infection [6]. In a retrospective cohort study by Lei et al., involving 34 surgical patients with confirmed COVID-19, the postoperative mortality rate was 20.5% [3]. Understanding post-infection recovery is therefore essential for enhancing postoperative care, disease management, long-term rehabilitation, optimizing healthcare resources, and shaping public health strategies [7]. However, patients with mild COVID-19 did not demonstrate an elevated risk of adverse postoperative outcomes at any assessed time point, and vaccination was associated with a reduced likelihood of mortality and other complications [8]. In certain cases, surgery must still be performed without delay. Nonetheless, limited research has examined COVID-19 patients who require ICU admission postoperatively.

This retrospective cohort study compared COVID-19 patients admitted to the ICU with those treated by the same surgical team at the Second Xiangya Hospital of Central South University. The primary endpoint was to assess surgical mortality and complication rates in COVID-19-positive patients relative to a closely matched control group. The secondary endpoint was to analyze the impact and risk factors related to postoperative pain.

## Methods

This study was approved by the Medical Ethics Committee of the Second Xiangya Hospital of Central South University (approval number: LYF20240197). Medical records were accessed between 8 October and 30 December 2024 for research purposes. The authors did not have access to any information that could identify individual participants during or after collection. As all participant identities were anonymized and there was no possibility of disclosing personal information, the requirement for written informed consent was waived by the institutional ethics committee. The date of first patient enrollment is December 1, 2022. Before patient enrollment, the study was prospectively registered at the Chinese Clinical Trial Register (ChiCTR2400089575, https://www.chictr.org.cn/showproj.html?proj=242638, Principal investigator: Ru-Yi Luo, registration date: 11 September 2024). This manuscript adheres to the applicable STROBE guidelines.

### Study participants

This retrospective cohort study was conducted at the Second Xiangya Hospital of Central South University, China, between December 2022 and January 2023. The study enrolled COVID-19-positive patients and COVID-19-negative controls, all of whom were admitted to the ICU following surgical treatment. Diagnosis of COVID-19 was based on nucleic acid testing using RT-PCR assays of nasopharyngeal swabs, which served as the gold standard for SARS-CoV-2 infection. Patients under 18 years of age, those with missing age information, and those who underwent gynecological, obstetrical, or transplant surgeries were excluded. Additionally, none of the included patients were critically ill or required intubation prior to surgery; all were admitted to the ICU postoperatively for monitoring. Due to the pandemic, only urgent or semi-urgent procedures were performed during the study period, and elective surgeries were largely suspended. The exclusion of pregnancy-related non-obstetric surgeries and transplant recipients aligns with standard perioperative research practices, as these groups differ substantially in clinical management and risk profiles [9,10].

**Fig 1:**
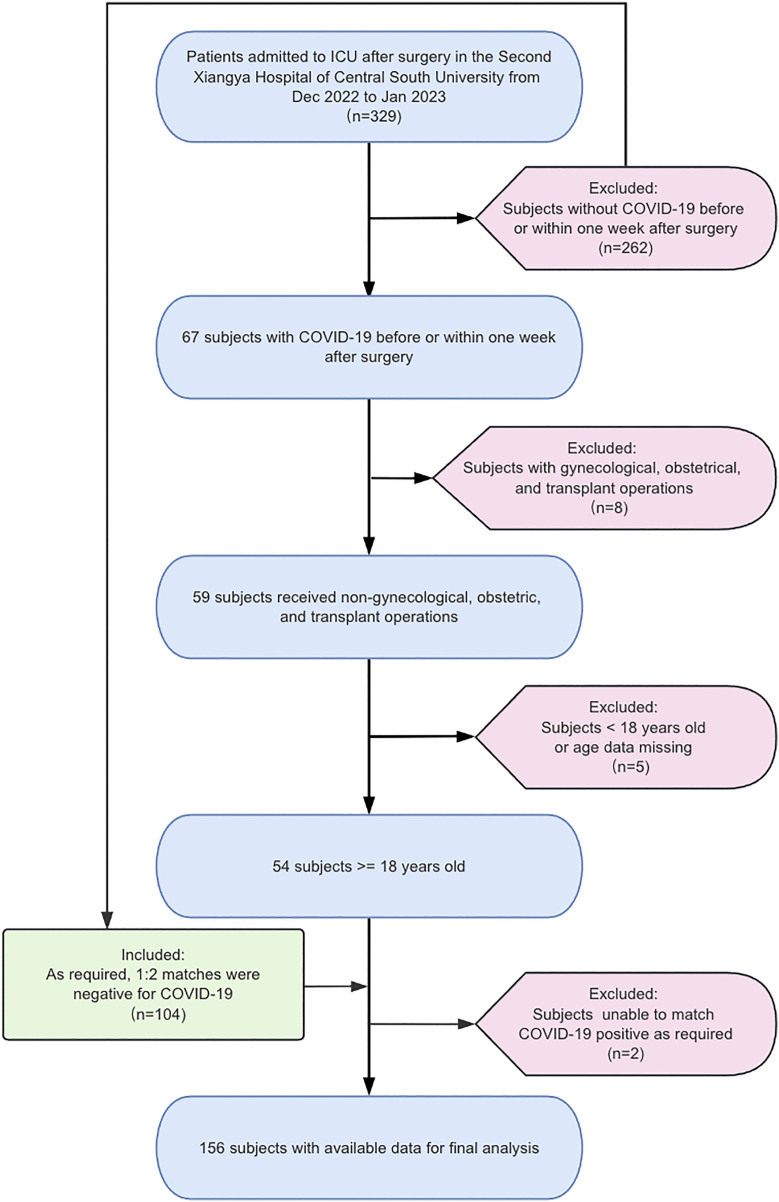
Schematic representation of the participant selection process and distribution of participant groups. Abbreviations: COVID-19, coronavirus disease 2019; ICU, Intensive Care Unit.

COVID-19-positive patients were matched in a 1:2 ratio to non-COVID-19 surgical ICU patients based on sex, age (within five years), and identical surgical pathology. Matching was performed according to COVID-19 testing results obtained within one week before or after surgery. Where possible, controls were selected from the same period; otherwise, the earliest eligible historical match was used. All patients were treated by the same medical team and received comparable care. A CONSORT-style flowchart summarizing patient selection and exclusion is provided in Fig 1.

### Study outcomes

The primary endpoint was to compare in-hospital surgical mortality and complication rates between COVID-19 patients and the closely matched control group. The secondary objective was to evaluate the impact of COVID-19 infection and identify risk factors associated with postoperative pain.

### Covariates

The following data were recorded for all COVID-19-affected patients admitted to the ICU after surgical treatment and their matched controls: sex, age, BMI, comorbidities (diabetes and hypertension), American Society of Anesthesiologists (ASA) class, and surgery type.

The descriptive statistics included key intraoperative variables such as duration of anesthesia, operative duration, transfusion, postoperative outcomes, hospitalization costs, and pain scale. Data were also collected on sepsis, sequential organ failure assessment (SOFA) score, hospital mortality or discontinued treatment, length of ICU and hospital stay, ventilator duration in the ICU, and the postoperative pain intensity. Pain intensity was assessed using the Visual Analog Scale (VAS), in which patients marked their perceived pain level on a 10-cm horizontal line anchored by “no pain” (0) and “worse pain imaginable” (10).

### Statistical analysis

The datasets included 156 patients, comprising 52 with COVID-19 (33.3%) and 104 closely matched controls (66.7%) across 17 variables. The Shapiro-Wilk test was conducted to assess whether continuous variables followed a normal distribution. Descriptive statistics were computed for the entire dataset, stratified by group (COVID-19 and Control) (Tables 1 and 2). The number of missing values was noted. The mean and standard deviation (SD) were reported for continuous variables with a normal distribution, and the Student's t-test was used for comparison. For continuous data that exhibited a skewed distribution, the median (interquartile range) and range (minimum-maximum) were presented, and the Mann-Whitney U test was employed for evaluation. Categorical or dichotomous variables were expressed as frequency or percentage, and comparisons were made using the Chi-square or Fisher’s exact test. Table 1 provides descriptive statistics on baseline characteristics used for matching, indicating that the matching was satisfactory for the reported features.

**Table 1:** Descriptive Statistics on Baseline Characteristics Used for Matching.

Variables	Total (n = 156)	Control (n = 104)	COVID-19 (n = 52)	P value
Sex, n (%)				
Male	105 (67.3)	70 (67.3)	35 (67.3)	1^a^
Female	51 (32.7)	34 (32.7)	17 (32.7)	
Age, year				
Mean ± SD	56.0 ± 12.1	56.0 ± 12.0	55.9 ± 12.4	0.944^b^
Age group, n (%)				
<65	114 (73.1)	77 (74)	37 (71.2)	0.702^a^
>=65	42 (26.9)	27 (26)	15 (28.8)	
BMI, kg/m^2^				
No. of any missing values	41	28	13	0.987^b^
Mean ± SD^†^	23.7 ± 3.6	23.7 ± 4.0	23.7 ± 2.7	
Diabetes, n (%)				
No	144 (92.3)	94 (90.4)	50 (96.2)	0.339^c^
Yes	12 (7.7)	10 (9.6)	2 (3.8)	
Hypertension, n (%)				
No	105 (67.3)	69 (66.3)	36 (69.2)	0.717^a^
Yes	51 (32.7)	35 (33.7)	16 (30.8)	
ASA class, n (%)				
Ⅱ	45 (28.8)	29 (27.9)	16 (30.8)	0.894^a^
Ⅲ	75 (48.1)	50 (48.1)	25 (48.1)	
Ⅳ	36 (23.1)	25 (24)	11 (21.2)	
Type of Surgery, n (%)				
Cardiothoracic	99 (63.5)	66 (63.5)	33 (63.5)	1^c^
Spinal	18 (11.5)	12 (11.5)	6 (11.5)	
Oral and maxillofacial	6 (3.8)	4 (3.8)	2 (3.8)	
Neurosurgery	15 (9.6)	10 (9.6)	5 (9.6)	
General	18 (11.5)	12 (11.5)	6 (11.5)	

Abbreviations: COVID-19, coronavirus disease 2019; SD: standard deviation; BMI (kg/m^2^), body mass index (calculated as weight in kilograms divided by height in meters squared); ASA, American Society of Anesthesiologists.

^a^Chi-square; ^b^t-test; ^c^Fisher’s exact test

**Table 2:** Descriptive Statistics of Intraoperative Features, Outcomes, Costs and Postoperative Pain in the Study Cohort.

Variables	Total (n = 156)	Control (n = 104)	COVID-19 (n = 52)	*P* value
Anesthesia duration, min				
Median (IQR) [range]	235.0 (160.0, 325.0) [65.0, 1235.0]	232.5 (155.0, 333.8) [65.0, 965.0]	242.5 (183.8, 310.0) [80.0, 1235.0]	0.973^b^
Operative duration, min				
Median (IQR) [range]	180.0 (123.8, 265.0) [30.0, 1150.0]	180.0 (120.0, 274.0) [30.0, 855.0]	180.0 (130.0, 228.8) [35.0, 1150.0]	0.933^b^
**Blood Transfusion**, n (%)				
No	132 (84.6)	93 (89.4)	39 (75.0)	**0.019** ^**a**^
Yes	24 (15.4)	11 (10.6)	13 (25.0)	
**Sepsis**, n (%)				
No	151 (96.8)	104 (100)	47 (90.4)	**0.004** ^**c**^
Yes	5 (3.2)	0 (0)	5 (9.6)	
**SOFA**				
Median (IQR) [range]	1.0 (0.0, 2.0) [0.0, 7.0]	1.0 (0.0, 2.0) [0.0, 5.0]	2.0 (0.8, 3.0) [0.0, 7.0]	**0.042** ^**b**^
**Postoperative ICU stay**, hour				
Median (IQR) [range]	24.0 (20.4, 64.8) [0.9, 960.0]	24.0 (19.9, 56.6) [0.9, 369.0]	40.8 (24.0, 85.4) [5.0, 960.0]	**0.013** ^**b**^
Postoperative hospital stay, days				
Median (IQR) [range]	9.0 (6.0, 14.0) [1.0, 49.0]	8.0 (5.8, 13.0) [2.0, 36.0]	9.0 (6.8, 14.5) [1.0, 49.0]	0.274^b^
**Hospital mortality or Discontinued treatment**, n (%)				
No	151 (96.8)	104 (100)	47 (90.4)	**0.004** ^**c**^
Yes	5 (3.2)	0 (0)	5 (9.6)	
Costs, 10,000 CNY				
Median (IQR) [range]	7.2 (4.6 12.5) [1.8, 77.8]	7.2 (4.6, 12.5) [1.8, 44.7]	7.5 (4.7, 12.3) [2.7, 77.8]	0.652^b^
**Ventilator duration in the ICU**, min				
Median (IQR) [range]	1014.0 (30.0, 1597.5) [0, 33120.0]	405.0 (27.0, 1387.5) [0, 8340.0]	1341.0 (510.0, 2110.5) [0, 33120.0]	**0.006** ^**b**^
VAS score				
Median (IQR) [range]	2.0 (1.0, 2.0) [0,6]	2.0 (1.0, 2.0) [0,5]	2.0 (2.0, 5.0) [0,6]	**<0.001** ^b^

Abbreviations: COVID-19, coronavirus disease 2019; IQR, interquartile range; SOFA: Sequential Organ Failure Assessment; ICU, Intensive Care Unit; CNY: Chinese Yuan; VAS, visual analog scale.

^a^Chi-square; ^b^Mann-Whitney U test; ^c^Fisher’s exact test

We calculated β coefficient and 95% confidence intervals (CI) through univariate and multivariate linear regression models to evaluate the association of COVID-19, treated as a categorical variable, with the VAS score for postoperative pain. Multiple imputation was performed to address the remaining missing data, with 30 imputed datasets created and analyzed collectively. All variables in the study were selected for model adjustments based on their statistical significance and clinical relevance. Model 1 was adjusted for sex, age and BMI. Model 2 included additional adjustments for diabetes and hypertension. Model 3 was further adjusted for intraoperative features, including type of surgery, ASA class, transfusion, ventilator duration in the ICU, anesthesia duration, and operative duration. Model 4 was adjusted for sepsis, SOFA score and hospital mortality or discontinued treatment. Model 5 was fully adjusted, similar to Model 4, with additional adjustments for postoperative ICU stay, postoperative hospital stay and hospitalization costs. Moreover, linear regression models were employed for interaction and subgroup analyses based on sex, age, and BMI.

All statistical analyses were conducted using R Statistical software (Version 4.2.2, The R Foundation) and the Free Statistics analysis platform (Version 1.9.2). A two-tailed *P* value <0.05 was considered statistically significant.

## Results

### Baseline characteristics of participants

The study included 52 patients diagnosed with COVID-19 out of 329 patients admitted to the ICU post-surgery, matched with 104 control subjects during the same study period from December 2022 to January 2023. Among these patients, the mean age was 56 years, and 105 individuals were male, constituting 67.3% of the sample (Table 1 and 2). Of the 52 COVID-19 patients, 19 (36.5%) contracted the virus within 7 days prior to surgery, while 33 (63.5%) were infected within 7 days following surgery (Fig 1). No significant differences were observed between the control and COVID-19 groups regarding sex, age, BMI, ASA class, comorbidities (diabetes and hypertension), or type of surgery (Table 1).

### Surgical management and anesthetic considerations in patients with COVID-19

Among patients with COVID-19, 33 out of 52 (63.5%) underwent cardiothoracic procedures. The remaining patients underwent spinal (12 [11.5%]), oral and maxillofacial (4 [3.8%]), general (12 [11.5%]), and neurosurgery (10 [9.6%]) procedures (Table 1). The mean operative duration and anesthesia duration did not differ significantly between the two groups (Table 2). However, the transfusion rate in the COVID-19 group was significantly higher than in the control group (10.6% vs. 25.0%, p = 0.019, Table 2). Moreover, the median SOFA score was 1.0 (IQR 0.0–2.0) in the overall cohort, with a statistically significant difference between the control group [1.0 (0.0–2.0)] and the COVID‑19 group [2.0 (0.8–3.0)] (P = 0.042).

### Death, complications, costs and postoperative pain in patients with COVID-19

Mortality rates were significantly higher in the COVID-19 cohort compared to the control group (5 patients [9.6%] vs. 0 patients [0%]; p = 0.004, Table 2). Among the five deceased patients in the COVID-19 group, causes of death included septic shock (n = 1), cardiac complications (n = 3), and disseminated intravascular coagulation (n = 1). In addition, the incidence of sepsis was markedly greater in the COVID-19 group than in the control group (Table 2). The COVID-19 cohort also exhibited a longer duration of mechanical ventilation and extended ICU stays compared to controls (Table 2). However, no statistically significant differences were observed in postoperative hospital stay or associated costs between the two groups.

Persistent pain is increasingly recognized as a significant manifestation of long COVID-19 [11,12]. Postoperative pain scores were compared between the COVID-19 and control groups, with the highest score within 7 days post-surgery selected for analysis. VAS scores were significantly higher in the COVID-19 group than in the control group (p < 0.001, Table 2), indicating that patients with perioperative COVID-19 infection experienced more severe acute postoperative pain.

### Associations between COVID-19 and VAS for pain

We conducted univariate and multivariate linear regression analyses to explore risk factors associated with postoperative pain. The results of the univariate analysis are presented in Table 3. Univariate linear regression analysis revealed that factors such as sex, age, BMI, diabetes, hypertension, type of surgery, ASA class, transfusion, ventilator duration in the ICU, anesthesia duration, operative duration, sepsis, SOFA score, postoperative ICU stay, hospital mortality or treatment discontinuation, and hospitalization costs were not significantly associated with the VAS score for postoperative pain. In contrast, COVID-19 infection and postoperative hospital stay were positively correlated with the VAS score.

**Table 3:** Univariate Linear regression Analyses of Postoperative Pain.

Variables	β Coefficient (95% CI)	*p* value (t-test)	*P* value (F-test)
**COVID-19: (+) vs (-)**	1.51 (1.08 ~ 1.94)	**< 0.001**	
Sex: Female vs Male	0.16 (−0.33 ~ 0.66)	0.521	
Age, year	0.004 (−0.02 ~ 0.02)	0.685	
BMI, kg/m^2^	0.004 (−0.08 ~ 0.07)	0.917	
Diabetes: Yes vs. No	0.03 (−0.85 ~ 0.90)	0.950	
Hypertension: Yes vs. No	−0.04 (−0.54 ~ 0.46)	0.868	
Type of Surgery: ref. = Cardiothoracic			0.212
Spinal	0.69 (−0.05 ~ 1.43)		
Oral and maxillofacial	−0.64 (−1.86 ~ 0.57)		
Neurosurgery	−0.14 (−0.94 ~ 0.66)		
General	0.36 (−0.38 ~ 1.1)		
ASA class: ref. = Ⅱ			0.146
Ⅲ	0.06 (−0.48 ~ 0.61)		
Ⅳ	−0.51 (−1.15 ~ 0.14)		
Blood Transfusion: Yes vs. No	0.03 (−0.62 ~ 0.68)	0.926	
**Ventilator duration in the ICU, min**	7e-05 (0 ~ 2e-04)	0.055	
Anesthesia duration, min	−0.001 (−0.003 ~ 2e-04)	0.092	
Operative duration, min	−0.001 (−0.003 ~ 1e-04)	0.073	
Sepsis: Yes vs. No	0.8 (−0.52,2.12)	0.232	
SOFA	0.04 (−0.12,0.2)	0.619	
Postoperative ICU stay, hour	0.002 (-3e-04 ~ 0.004)	0.080	
**Postoperative hospital stay**, days	0.03 (0 ~ 0.06)	**0.045**	
Hospital mortality or Discontinued treatment: Yes vs. No	0.18 (−1.14 ~ 1.51)	0.787	
Costs, 10,000 CNY	-2e-04 (−0.002 ~ 0.002)	0.834	

Abbreviations: 95% CI, 95% confidence intervals; COVID-19, coronavirus disease 2019; BMI (kg/m^2^), body mass index (calculated as weight in kilograms divided by height in meters squared); ref., reference; ASA, American Society of Anesthesiologists; SOFA: Sequential Organ Failure Assessment; ICU, Intensive Care Unit; CNY: Chinese Yuan.

Furthermore, we performed multivariable linear regression to assess the association between COVID-19 infection and the VAS score for postoperative pain, as shown in Table 4. The initial analysis using the unadjusted crude model indicated a significant positive association (β = 1.51; 95% CI: 1.08 ~ 1.94; p < 0.001). Covariates were then incrementally introduced across five adjusted models. The β values for the association between COVID-19 infection and VAS score in these adjusted models were 1.51 (95% CI: 1.08 ~ 1.94), 1.52 (95% CI: 1.09 ~ 1.96), 1.41 (95% CI: 0.96 ~ 1.86), 1.57 (95% CI: 1.10 ~ 2.03), and 1.51 (95% CI: 1.03 ~ 1.98). The *P* values in all five models were less than 0.001, indicating a consistent and statistically significant association.

**Table 4:** Multivariate linear regression analyses at multiple interpolation of Postoperative Pain.

Variable	COVID-19 (n = 156)			
	before multiple interpolation		after multiple interpolation	
	β (95% CI)	*p* value	β (95% CI)	*p* value
Unadjusted	1.51 (1.08 ~ 1.94)	<0.001	1.51 (1.08 ~ 1.94)	<0.001
Model 1	1.43 (0.95 ~ 1.92)	<0.001	1.51 (1.08 ~ 1.94)	<0.001
Model 2	1.44 (0.95 ~ 1.93)	<0.001	1.52 (1.09 ~ 1.96)	<0.001
Model 3	1.27 (0.75 ~ 1.78)	<0.001	1.41 (0.96 ~ 1.86)	<0.001
Model 4	1.42 (0.88 ~ 1.96)	<0.001	1.57 (1.10 ~ 2.03)	<0.001
Model 5	1.36 (0.83 ~ 1.90)	<0.001	1.51 (1.03 ~ 1.98)	<0.001

Model 1: adjusted for sociodemographic variables (sex, age, BMI).

Model 2: adjusted for model 1 + diabetes + hypertension.

Model 3: adjusted for model 2 + ASA class + type of surgery + transfusion + ventilator duration in the ICU + anesthesia duration + operative duration.

Model 4: adjusted for model 3 + sepsis + SOFA score + hospital mortality/discontinued treatment.

Model 5: adjusted for model 4 + postoperative ICU stay + postoperative hospital stay + costs.

Abbreviations: COVID-19, coronavirus disease 2019; 95%CI, 95% confidence intervals; BMI, body mass index (calculated as weight in kilograms divided by height in meters squared); ASA, American Society of Anesthesiologists; ICU, Intensive Care Unit; SOFA: Sequential Organ Failure Assessment.

To eliminate the influence of preoperative and postoperative COVID-19 infection on postoperative pain, we categorized the COVID-19 group into preoperative and postoperative infection subgroups. Multivariate linear regression analysis was performed on data from these two subgroups. The results revealed no statistically significant differences between them (S1 Table), suggesting that the timing of infection did not significantly affect the postoperative pain scores.

**Fig 2:**
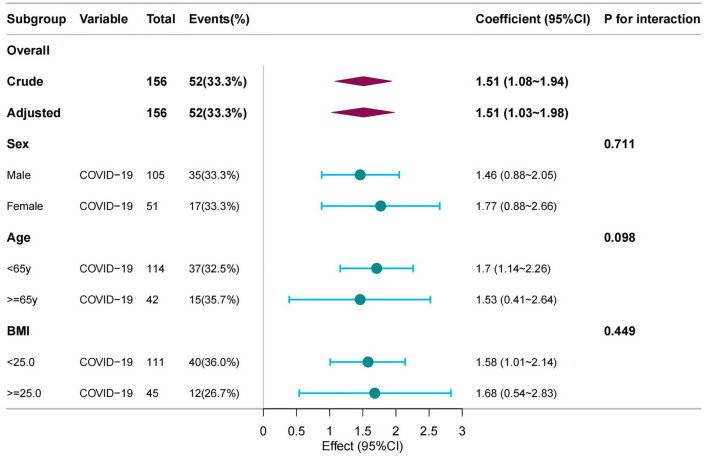
Association between COVID-19 and VAS score for postoperative pain in different subgroups. Abbreviations: BMI, body mass index (calculated as weight in kilograms divided by height in meters squared); COVID-19, coronavirus disease 2019; VAS, visual analog scale; y, years; 95% CI: 95% confidence intervals.

We further performed stratified and interaction analyses by sex, age, and BMI to determine whether the association between COVID-19 infection and VAS score for postoperative pain was consistent across different subgroups (Fig 2). The stratified analysis indicated that the results across all subgroups were consistent with the multivariate linear regression findings. No statistically significant interactions were detected in the stratified analyses, suggesting no effect modification.

## Discussion

### Primary findings

Surgical procedures in patients with COVID-19 were associated with increased rates of blood transfusions, sepsis and hospital mortality, as well as higher SOFA score, longer durations of mechanical ventilation and extended stays in the ICU. Moreover, COVID-19 infection was linked to elevated postoperative pain scores. While the 1.51-unit increase in VAS score was statistically significant, its clinical significance—for example, its impact on functional recovery, opioid consumption, or patient-reported quality of life—requires further investigation in future studies specifically designed to assess these outcomes. Collectively, these findings highlight the need for tailored postoperative care and pain management strategies in this patient population.

Postoperative pulmonary complications have been reported in approximately 50% of patients with perioperative COVID-19 infection, significantly correlating with increased mortality rates [13]. Consequently, it is recommended to defer non-urgent surgical procedures and prioritize non-operative treatment options when feasible [13]. A Canadian cohort study reported a 30-day postoperative mortality rate of 15.9% among COVID-19 patients undergoing surgery [14]. Our observed mortality rate of 9.6%, although lower than early pandemic reports, remains substantially higher than in matched controls, underscoring a significant perioperative risk. This may reflect the characteristics of our ICU-admitted cohort, the influence of SARS-CoV-2 variants prevalent during late 2022, or the potential impact of prior vaccination and improved treatment protocols, which were not assessed in this study. Consistently, our research demonstrated that surgical patients with COVID-19 experienced higher incidences of blood transfusions, sepsis, mortality, and longer durations of mechanical ventilation while in the ICU, as well as extended ICU stays. These findings suggest that anesthesiologists and surgeons should enhance preoperative assessments and postoperative surveillance, optimize surgical timing, implement personalized treatment protocols, and adopt a multidisciplinary approach to perioperative care to reduce mortality and improve outcomes.

The COVID-19 pandemic has also been associated with new-onset chronic pain, particularly among patients requiring intensive support [11]. Headache is often an initial symptom and may correlate with a milder disease trajectory during the acute phase. The association between COVID-19 and increased postoperative pain observed in our study may be driven by several mechanisms. The systemic hyperinflammatory and pro-thrombotic state induced by COVID-19 may exacerbate surgical stress and impair tissue healing. In addition, SARS-CoV-2 may have neurotropic effects, potentially altering peripheral and central pain processing pathways and increasing postoperative pain sensitization. Despite increasing awareness of pain management as a critical aspect of care, limited literature addresses postoperative pain in COVID-19 patients. Pain is recognized as the fifth vital sign [15], and effective pain control is crucial for postoperative rehabilitation and enhanced recovery [16]. Our findings indicate that COVID-19 infection can increase postoperative pain by 1.51 units, independent of sex, age, and BMI.

Poorly managed postoperative pain can contribute to chronic pain development, with studies reporting that up to 40% of patients in UK pain clinics attributed their chronic pain to surgical or traumatic injuries [17]. Pain is also a risk factor for postoperative cognitive dysfunction (POCD), likely due to overlapping neural substrates for pain perception and cognitive control [18]. Additionally, increased postoperative pain can lead to pulmonary complications such as atelectasis and pneumonia due to restricted breathing and impaired coughing [19]. These complications can prolong hospitalization, increase readmission rates, and elevate healthcare resource utilization, thereby increasing economic burdens on patients and healthcare systems [20]. The elevated pain scores in COVID-19 patients highlight a critical area for intervention, as effective pain management may mitigate adverse health outcomes and reduce associated costs. Patients with COVID-19 should therefore exercise caution when undergoing surgical procedures.

Our findings also demonstrated that COVID-19 patients experienced higher rates of blood transfusion, sepsis, and hospital mortality, likely linked to the profound inflammatory and pro-thrombotic state induced by SARS-CoV-2 infection. COVID-19 is associated with coagulopathy, characterized by thrombotic and hemorrhagic complications, which may explain the increased transfusion requirements in our cohort [21,22]. The virus also induces lymphopenia and immune dysfunction, impairing the host's ability to combat secondary bacterial infections and predisposing patients to sepsis [23,24]. This immune dysregulation, together with surgical stress, likely contributes to elevated mortality. Our results align with existing literature identifying immune and coagulation dysfunction as key predictors of poor outcomes in hospitalized COVID-19 patients and establish perioperative COVID-19 as an independent risk factor for adverse events. These findings underscore the importance of enhance preoperative assessments and postoperative surveillance for these complications in COVID-19 patients.

In this study, five patients died postoperatively. One patient died of septic shock following surgery for a perforated sigmoid colon, another from low cardiac output syndrome after cardiac surgery, and a third, admitted with severe hemorrhagic shock, died due to disseminated intravascular coagulation (DIC). In another case, a patient with acute respiratory distress syndrome (ARDS) and multiple comorbidities had ventilator support withdrawn for financial reasons, leading to death. Finally, a polytrauma patient undergoing spinal surgery with a history of atrial fibrillation, coronary artery disease, and renal failure died from postoperative cardiac complications. All deceased patients had preexisting comorbidities and underwent emergency surgery for acute hemorrhage or intestinal perforation. Deaths were attributed to postoperative sepsis or exacerbation of underlying conditions.

### Limitations

This study has several limitations that should be acknowledged. First, its retrospective, single-center design, conducted during a specific phase of the pandemic, may introduce selection and information biases and limits the generalizability of the findings to other settings, populations, or future SARS-CoV-2 variants. Second, the relatively small sample size of ICU-admitted surgical patients with COVID-19 restricts statistical power for detailed subgroup analyses and for examining potential dose-response relationships. This focused cohort also represents a more severe spectrum of illness, which may not be generalizable to all surgical patients with perioperative COVID-19 infection, particularly those with mild or asymptomatic disease. Third, despite rigorous matching, unmeasured or inconsistently documented variables—such as COVID-19 severity, viral load, duration of symptoms, specific respiratory parameters, vaccination status, SARS-CoV-2 variant, and comorbidities including malignancy or chronic immunosuppression—may lead to residual confounding. Fourth, postoperative pain assessment based on the peak VAS score within seven days may not fully capture temporal fluctuations or the modulating effects of analgesic regimens over time. Finally, as an observational study, it can establish associations but not causality. Future prospective, multi-center studies with larger cohorts and standardized data collection are needed to validate these findings and further elucidate the underlying mechanisms.

## Conclusions

This study demonstrated that surgical patients with COVID-19 faced increased risks of blood transfusion, postoperative infection, mortality, prolonged mechanical ventilation in the ICU, and extended ICU stays, along with higher postoperative pain levels. Although causality cannot be inferred, the consistent associations observed across adjusted models reinforce these findings. These results emphasize the need for vigilant, individualized perioperative care for this vulnerable population. We recommend enhanced preoperative optimization, careful intraoperative management, and proactive multimodal analgesia for surgical patients with perioperative COVID-19. Future large-scale, prospective studies with detailed phenotyping are warranted to confirm these associations, elucidate the underlying mechanisms, and evaluate tailored perioperative care bundles aimed at mitigating these risks.

## Supporting information

S1 Table Multivariable linear regression analyses at multiple interpolation of Postoperative Pain. (DOCX)
