# Supplementary material for: Influence of COVID-19 on postoperative prognosis and pain management
Source: PLoS One. 2026 Mar 9;21(3):e0344211. doi: 10.1371/journal.pone.0344211 (PMC12970926; doi:10.1371/journal.pone.0344211)
Supplement: S1 Table — (DOCX) [file pone.0344211.s001.docx]

**Supplemental** **Table S1. Multivariable linear regression analyses at multiple interpolation of Postoperative Pain**

| Variable | Covid-19^*^ (n=52) | | | | |
| --- | --- | --- | --- | --- | --- |
|  | before multiple interpolation | |  | after multiple interpolation | |
|  | β (95% CI^†^) | p value |  | β (95% CI^†^) | p value |
| Unadjusted | -0.38 (-1.42~0.65) | 0.471 |  | -0.38 (-1.42~0.65) | 0.471 |
| Model 1 | -0.25 (-1.41~0.91) | 0.671 |  | -0.52 (-1.63~0.59) | 0.363 |
| Model 2 | -0.34 (-1.54~0.85) | 0.580 |  | -0.59 (-1.73~0.55) | 0.319 |
| Model 3 | -0.39 (-1.83~1.06) | 0.606 |  | -0.08 (-1.57~1.40) | 0.912 |
| Model 4 | -0.20 (-1.67~1.27) | 0.796 |  | -0.07 (-1.55~1.41) | 0.927 |
| Model 5 | -0.03 (-1.56~1.50) | 0.971 |  | 0.30 (-1.20~1.80) | 0.698 |

Model 1: adjusted for sociodemographic variables (sex, age, BMI^‡^).

Model 2: adjusted for model 1 + diabetes + hypertension.

Model 3: adjusted for model 2 + type of surgery + ASA^§^ class + transfusion + ventilator duration + type of anesthesia + anesthesia duration + operative time.

Model 4: adjusted for model 3 + sepsis + hospital mortality/discontinued treatment.

Model 5: adjusted for model 4 + postoperative ICU^⁑^ stay + postoperative hospital stays + costs.

Abbreviations: *COVID-19, coronavirus disease 2019; †95% CI, 95% confidence intervals; ‡BMI, body mass index (calculated as weight in kilograms divided by height in meters squared); §ASA, American Society of Anesthesiologists; ⁑ICU, Intensive Care Unit.
